# 基于特征肽的超高效液相色谱-串联质谱法检测矛头蝮蛇蛇毒种属来源及类凝血酶含量

**DOI:** 10.3724/SP.J.1123.2021.12020

**Published:** 2022-09-08

**Authors:** Ruiqing XIAN, Baojian HANG, Liping GONG, Congcong WANG, Xunjie ZHANG, Li PENG, Feng SHI

**Affiliations:** 1.山东省食品药品检验研究院, 国家药品监督管理局仿制药研究与评价重点实验室, 山东 济南 250101; 1. National Medical Products Administration (NMPA) Key Laboratory for Research and Evaluation of Genetic Drugs, Shandong Institute for Food and Drug Control, Jinan 250101, China; 2.山东大学药学院, 山东 济南 250012; 2. School of Pharmaceutical Sciences, Shandong University, Jinan 250012, China; 3.华润双鹤利民药业(济南)有限公司质量部, 山东 济南 250200; 3. Department of Quality Control, China Resources Double-Crane Limin Pharmaceutical (Jinan) Co., Ltd., Jinan 250200, China

**Keywords:** 超高效液相色谱-串联质谱, 特征肽, 蛇毒类凝血酶, 矛头蝮蛇, 蛇毒, ultra-high performance liquid chromatography-tandem mass spectrometry (UHPLC-MS/MS), marker peptide, snake venom thrombin-like enzymes (svTLEs), *Bothrops atrox*, snake venom

## Abstract

蛇毒血凝酶类药物是以蝮蛇蛇毒为原料制备的止血药,主要活性成分为蛇毒类凝血酶(svTLEs)。不同蛇种来源的svTLEs结构不同,止血机制不同,药理作用也存在差异,因此准确鉴别蛇毒种属来源和svTLEs含量对于保障该类产品的质量至关重要。研究基于蛋白质组学技术,筛选出了具有种属特异性的矛头蝮蛇svTLE特征肽,并建立了基于特征肽的超高效液相色谱-串联质谱(UHPLC-MS/MS)检测矛头蝮蛇蛇毒种属来源及类凝血酶含量的方法。采用胰蛋白酶对纯化的矛头蝮蛇svTLE进行酶解,利用纳升液相色谱-四极杆/静电场轨道阱高分辨质谱(Nano LC-Q-Exactive-MS)和Proteome Discoverer 2.2软件分别进行多肽的检测和鉴定,通过BLAST搜索与Uniprot数据库对比分析,筛选出具有种属特异性的矛头蝮蛇svTLEs特征肽“EAYNGLPAK”。针对该特征肽对酶解温度、酶解时间和酶用量等样品前处理方法进行了优化,利用超高效液相色谱-串联质谱,以*m/z* 481.9>315.2和481.9>485.2作为检测离子对,采用ESI^+^模式进行了多反应监测(MRM)定性定量分析。结果显示,特征肽在2.5~30 ng/mL范围内线性关系良好,相关系数(*r*)大于0.9996,多水平加标回收率范围为95.5%~101.9%,各水平平行测定结果的相对标准偏差(RSD)为1.1%~3.2%,完全能够满足实际样品检测需求。方法简便快捷,灵敏度高,专属性强,可用于矛头蝮蛇蛇毒种属鉴别及svTLE含量测定,从源头保证血凝酶类产品的质量,并可为其他蛇毒类产品的质量控制提供参考。

## Abstract (en)

Snake venom thrombin drugs are hemostatic drugs prepared from *Agkistrodon halys* venom, and the main active ingredients are snake venom thrombin-like enzymes (svTLEs). The svTLEs derived from different snake species differ in their structures, hemostatic mechanisms, and pharmacological effects. Therefore, accurate identification of the source of snake venom species and determination of the svTLE content are essential to ensure the quality of these products. Based on proteomics technology, the marker peptides of svTLEs from *Bothrops atrox* were screened with species specificity for the first time in this study, and an ultra-high performance liquid chromatography-tandem mass spectrometry (UHPLC-MS/MS) method for species identification and determination of the svTLE content of *Bothrops atrox* was established. After reductive alkylation and trypsin enzymolysis of the purified svTLE from *Bothrops atrox*, enzymatic peptide fragments were obtained and determined by easy-nano liquid chromatography-quadrupole/electrostatic field orbitrap high resolution mass spectrometry (Nano LC-Q-Exactive-MS). The mass spectrum data were analyzed by Proteome Discoverer 2.2 software. The maker peptide “EAYNGLPAK”, which characterized the svTLE from *Bothrops atrox*, was finally screened and validated by comparison of the basic local alignment search tool (BLAST) with the NCBI and UniProt databases. For the marker peptide, the enzymolysis temperature, enzymolysis time and amount of enzyme for the sample preparation were optimized. The optimized enzymolysis conditions were as follows: enzymolysis temperature, 37 ℃; enzymolysis time, 4 h; and amount of enzyme, 10 μL. A qualitative and quantitative detection method based on UHPLC-MS/MS was established by optimizing the chromatographic and mass spectrometric conditions. Accordingly, 20 mg of the evenly mixed sample was weighed and placed in a 100 mL volumetric flask. Then, 25 mmol/L ammonium bicarbonate solution was added to dissolve the sample, and the solution was diluted to the scale. Precisely 1.00 mL of the solution was extracted; subsequently, addition of 10 μL trypsin solution was added, followed by shaking, and the mixture was placed in an incubator for 4 h to induce enzymolysis at a constant temperature of 37 ℃. The mixture was subsequently removed from the incubator, cooled to ambient temperature, centrifuged at 12000 r/min for 10 min, and analyzed by LC-MS. Separation was performed on the UPLC system with a Thermo Hypersil GOLD C18 column (100 mm×2.1 mm, 3.0 μm) under the gradient elution of acetonitrile containing 0.1% (v/v) acetic acid and water containing 0.1%(v/v) acetic acid, at a flow rate of 0.3 mL/min, column temperature of 30 ℃, and injection volume of 2 μL. The maker peptides were determined in the electrospray positive ionization (ESI^+^) and multiple reaction monitoring (MRM) modes using the external standard curve method. The detection ions were *m/z* 481.9> 315.2 and 481.9> 485.2. There was a good linear relationship between the mass concentration of the marker peptide and the chromatographic peak area in the range of 2.5-30 ng/mL, and the correlation coefficient (*r*) was 0.9996, The limit of detection (*S/N*=3) and limit of quantification (*S/N*=10) were 2.5 mg/kg and 6.25 mg/kg, respectively. At spiked levels of 40, 80, and 120 mg/kg, the recoveries of the marker peptides were 95.5%-101.9%, while the relative standard deviations (RSDs) of the results for parallel analyses at various spiked levels were 1.1%-3.2%. The developed method is simple, rapid, sensitive, and specific, and it can be used for the identification of *Bothrops atrox* venom species and determination of the svTLE content. The findings of this study would help ensure the quality of hemocoagulase products from the relevant source and provide a reference for the quality control of other snake venom products.

蛇毒血凝酶类药物是一类以蝮蛇蛇毒为原料制备的止血药,主要活性成分为蛇毒类凝血酶(svTLEs),其在止血领域中占有重要地位,占止血药市场份额近50%^[1,2]^。目前,已上市蛇毒血凝酶类药物主要来源于矛头蝮蛇、尖吻蝮蛇、蝰蛇和白眉蝮蛇等蛇种,不同蛇种来源的类凝血酶结构不同,其作用机制不同,相应的药理作用也存在差异^[3,4]^,因此准确鉴别蛇毒种属来源和svTLEs含量对于保障该类产品的质量至关重要。

现报道的蛇毒种属鉴别及蛋白质检测方法主要为酶联免疫吸附法(ELISA)^[5]^、液相色谱法(LC)^[6]^和电泳法等^[6]^,存在方法繁琐、灵敏度低、专属性差等问题。伴随着生物质谱技术的飞速发展,基于质谱的蛋白质组学研究日渐成熟,以肽生物标志物为基础的蛋白质组学技术为食品药品质量控制提供了一种新思路,已成功应用于肉类产品成分鉴定、中药掺伪筛查等方面^[7⇓⇓⇓⇓⇓⇓⇓⇓⇓-17]^,但在生化药物领域研究较少,尚未见基于特征肽的蛇毒类凝血定性定量研究报道。

本研究采用纳升液相色谱-四极杆/静电场轨道阱高分辨质谱(Nano LC-Q-Exactive-MS)筛选了矛头蝮蛇类凝血酶特征肽,并采用超高效液相色谱-串联质谱(UHPLC-MS/MS)建立了检测矛头蝮蛇蛇毒种属来源及类凝血酶含量的方法。与现有检测方法相比,本研究所建立的方法分析时间短,专属性更强,灵敏度更高,为从源头保证矛头蝮蛇蛇毒类产品的质量及用药安全性提供了技术支持,并可为其他蛇毒类产品的质量控制提供参考。

## 1 实验部分

### 1.1 仪器与试剂

EASY-nLC 1000纳升液相色谱联用Q-Exactive高分辨质谱(美国Thermo Scientific公司), Triple Quad 6500+型超高效液相色谱-三重四极杆质谱联用仪(美国AB Sciex公司), CP225D电子天平(德国Sartorius公司), Sigma 3-30 K冷冻离心机(德国Sigma公司), Milli-Q Advantage A10超纯水仪(美国Millipore公司)。

乙腈(色谱纯,美国Honeywell公司),甲酸(色谱纯,美国ACS恩科化学公司),胰蛋白酶(序列分析纯,德国Sigma公司),碘乙酰胺(分析纯,德国Sigma公司),三羟甲基氨基甲烷(分析纯,天津市科密欧化学试剂有限公司),二硫苏糖醇(纯度≥99%,上海翌圣生物科技有限公司),特征肽EAYNGLPAK(纯度≥98%,上海强耀生物科技有限公司)。

蛇毒SV1~SV3、矛头蝮蛇类凝血酶(纯度98.5%)(蓬莱诺康药业有限公司),蛇毒SV4~SV6(锦州奥鸿药业有限责任公司)。

### 1.2 Nano LC-Q-Exactive-MS条件

采用Thermo ReproSil-Pur C18-AQ Trap柱(35 mm×0.2 mm, 5 μm)脱盐富集,采用Thermo ReproSil-Pur C18-AQ纳升分析柱(250 mm×75 μm, 3 μm)进行分离。流动相A: 0.1%甲酸水-乙腈(98∶2, v/v);流动相B: 0.1%甲酸水-乙腈(2∶98, v/v)。纳升分离泵流速:300 nL/min。梯度洗脱程序:0~5.0 min, 100%A; 5.0~20.0 min, 100%A~90%A; 20.0~75.0 min, 90%A~68%A; 75.0~95.0 min, 68%A~50%A; 95.0~100.0 min, 50%A~100%A。

电喷雾电离(ESI)源,正离子模式,喷雾电压为2.0 kV,离子传输毛细管温度为275 ℃,离子传输透镜(S-Lens)传输效率设置为60%。一级质谱采用静电场轨道阱(Orbitrap)作为质量分析器,分辨率为60000,采集范围为350~1650。二级质谱采用Top20数据依赖模式进行母离子选择,采用高能诱导裂解(HCD)模式进行碎裂,归一化碰撞能量(NCE)设置为35%。

### 1.3 UHPLC-MS/MS条件

色谱柱:Thermo Hypersil GOLD C18色谱柱(100 mm×2.1 mm, 3 μm);柱温:40 ℃;流速:0.3 mL/min;流动相A: 0.1%甲酸水溶液,流动相B: 0.1%甲酸乙腈。梯度洗脱程序:0~2.0 min, 10%B; 2.0~6.0 min, 10%B~40%B; 6.0~6.1 min, 40%B~70%B; 6.1~8.0 min, 70%B; 8.0~8.1 min, 70%B~10%B; 8.1~10.0 min, 10%B。进样量:10 μL。

ESI源,正离子扫描模式,多反应监测(MRM)模式;离子源温度:500 ℃。定性、定量离子对(双电荷)、锥孔电压、碰撞能量见表1。

**表 1:** 特征肽的质谱采集离子信息

Marker peptide	Parent ion (m/z)	Product ion (m/z)	Cone voltage/V	Collision energy/eV
EAYNGLPAK	481.9	315.2^*^	80	22
		485.2	80	20

* Quantitative ion.

### 1.4 溶液的制备

#### 1.4.1 特征肽筛选溶液

取矛头蝮蛇类凝血酶5 mg,置于10 mL量瓶中,加25 mmol/L碳酸氢铵溶液溶解并定容;精密量取200 μL,加0.2 mol/L二硫苏糖醇溶液10 μL,混匀,60 ℃反应1 h,加0.2 mol/L碘乙酰胺溶液20 μL,避光放置30 min,加25 mmol/L碳酸氢铵溶液760 μL和10 mg/mL胰蛋白酶溶液10 μL, 37 ℃反应90 min, 90 ℃灭活10 min,放冷至室温,12000 r/min离心10 min,取上清液作为特征肽筛选用溶液,按1.2节条件进行检测。

#### 1.4.2 特征肽对照品储备液

取约10 mg特征肽对照品,精密称定,置于100 mL量瓶中,加25 mmol/L碳酸氢铵溶液并定容至刻度,摇匀,精密量取上述溶液(100 μg/mL)适量,用25 mmol/L碳酸氢铵溶液稀释制得质量浓度为100 ng/mL特征肽对照品储备液。

#### 1.4.3 特征肽系列标准溶液

取1.4.2节特征肽对照品储备液适量,用25 mmol/L碳酸氢铵溶液稀释制得质量浓度分别为2.5、5、8、10、12、20和30 ng/mL的系列标准溶液,按1.3节条件进行检测。

#### 1.4.4 供试品溶液

取供试品20 mg,置于100 mL容量瓶中,然后加入浓度为25 mmol/L的碳酸氢铵溶液定容,摇匀;精密量取上述溶液1 mL,加入质量浓度为10 mg/mL的胰蛋白酶溶液10 μL,在37 ℃下反应4 h, 12000 r/min离心10 min,取上清液作为供试品溶液,按1.3节条件进行检测。

#### 1.4.5 空白溶液

精密量取浓度为25 mmol/L的碳酸氢铵溶液1 mL,加入10 mg/mL胰蛋白酶溶液10 μL, 37 ℃下反应4 h, 12000 r/min离心10 min,取上清液作为空白溶液,按1.3节条件进行检测。

## 2 结果与讨论

### 2.1 实验条件考察

#### 2.1.1 特征肽的筛选

特征肽的选择是开发液相色谱-串联质谱法,进行蛋白质定性定量分析的关键和难点,直接决定了后续建立方法的特异性、灵敏度和稳定性。本研究采用普适性强的酶解条件,对纯化的矛头蝮蛇类凝血酶进行酶解,以获得丰富的酶解肽段,并采用纳升液相色谱-串联高分辨质谱进行分析,采集了高质量的质谱数据。使用Proteome Discoverer 2.2软件(美国Thermo Scientific公司)对质谱数据进行分析,初步筛选出了矛头蝮蛇类凝血酶的备用多肽序列EAYNGLPAK、NVITDKDIMLIR、FICPN、KNVITDKDIMLIR,进一步参考特征肽筛选一般原则^[18]^,选择“EAYNGLPAK”作为矛头蝮蛇类凝血酶的特征肽。为进一步验证该特征肽的种属特异性,在NCBI数据库里对特征肽“EAYNGLPAK”进行BLAST比对分析,确认其为矛头蝮蛇毒类凝血酶特有,具有种属特异性。根据NCBI数据库中矛头蝮蛇类凝血氨基酸序列(UniProtKB/Swiss-Prot: P04971.1)可知,其序列中仅存在唯一的EAYNGLPAK肽段,可以直接用特征肽的含量表征矛头蝮蛇蛇毒中类凝血酶的含量。该特征肽的一级和二级质谱如图1所示。

**图 1:**
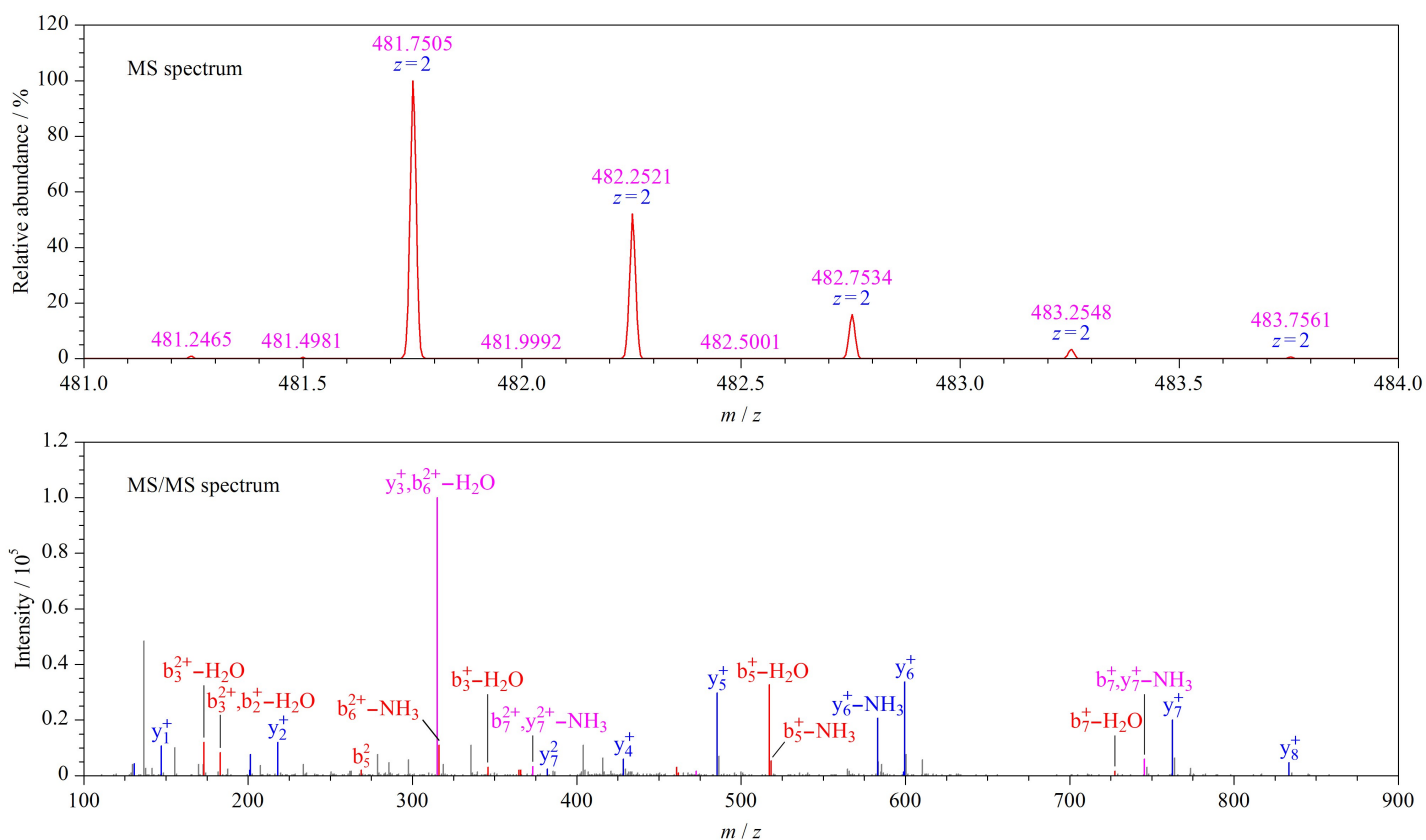
特征肽(EAYNGLPAK)的一级和二级质谱图

#### 2.1.2 特征肽MS/MS参数的优化

为保证方法的实用性和可推广性,本研究采用MRM模式监测特征肽的碎片离子,以实现复杂基质中目标蛋白质的精确定量。为获得合适的离子对和质谱采集参数,提高检测灵敏度和特异性,本实验采用蠕动泵(流速10 μL/min)向三重四极杆质谱中引入人工合成特征肽(EAYNGLPAK)标准溶液(100 ng/mL)。通过一级全扫描找到特征肽的母离子,再分别以一级母离子通过二级全扫描找到二级碎片离子,并通过不断改变碰撞能使碎片离子响应增强,通过优化获得最佳离子源参数,确定锥孔电压及碰撞能量。

#### 2.1.3 酶解条件的优化

二硫键还原条件的考察:固定酶解温度(37 ℃)、酶用量(10 μL)、酶解时间(4 h),考察了二硫苏糖醇和碘乙酰胺对特征肽量的影响。结果显示,酶解体系中是否加入二硫苏糖醇和碘乙酰胺对产生的特征肽量无显著影响,因此选择无还原剂的直接酶解条件。

酶解时间的考察:固定酶解温度(37 ℃)、酶用量(10 μL),考察酶解时间对测定结果的影响。比较了不同酶解时间(0.5、1、2、4、6、8、12 h)产生的特征肽峰面积。由图2可以看出,随着酶解时间的延长,特征肽的含量逐渐升高,当酶解时间超过4 h以后,结果趋于稳定,说明酶解反应趋于完全,因此本方法选择酶解时间为4 h。

**图 2:**
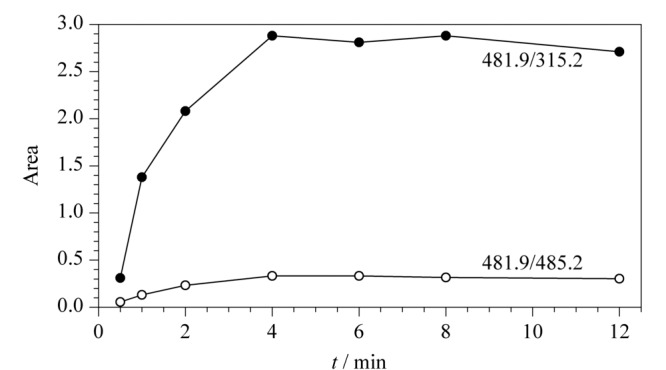
不同酶解时间下监测离子的峰面积

酶的用量:固定酶解温度(37 ℃)和酶解时间(4 h),考察酶的用量对测定结果的影响。由图3可以看出,在胰蛋白酶质量浓度为10 mg/mL时,酶的用量超过10 μL以后,结果趋于稳定,说明当酶的用量为10 μL时,足以完全酶解样品,因此本方法选择酶的用量为10 μL。

**图 3:**
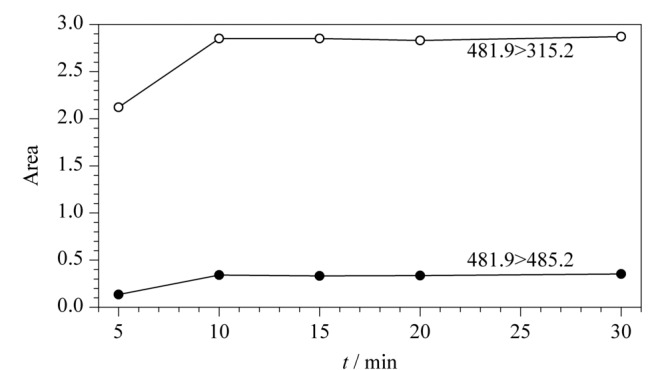
不同酶用量下监测离子的峰面积

### 2.2 方法学验证

#### 2.2.1 专属性

取1.4节制备的特征肽对照品溶液(10 ng/mL)、空白溶液及阴性样品溶液(取SV3蛇毒样品按1.4.4节方法制备),按1.3节UHPLC-MS/MS进行分析。结果显示空白溶液和阴性样品溶液在特征肽对照品溶液出峰位置无干扰峰出现,特征肽峰形良好,MRM色谱图见图4,表明该方法专属性良好。

**图 4:**
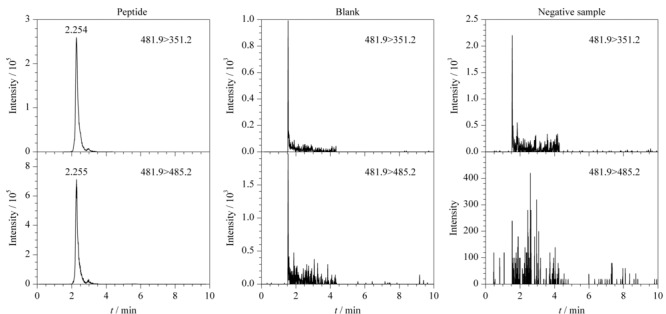
特征肽对照品溶液(10 ng/mL)、空白溶液及阴性样品溶液的MRM色谱图

#### 2.2.2 标准曲线与线性范围

取系列标准溶液,按1.3节UHPLC-MS/MS条件采集质谱数据。以定量离子对*m/z* 481.9>315.2的峰面积为纵坐标(*y*),以对照品溶液中特征肽的质量浓度为横坐标(*x*, ng/mL),绘制标准曲线,得到线性回归方程*y*=143868*x*-54779(*r*=0.9996),在2~30 ng/mL范围内,特征肽的质量浓度与色谱峰面积呈良好的线性关系。

#### 2.2.3 检出限与定量限

取1.4.3节2.5 ng/mL的特征肽对照品溶液适量,用1.4.5节的空白溶液稀释制得不同浓度的特征肽溶液,进样测定。当特征肽质量浓度为0.5 ng/mL和1.25 ng/mL时,对应的峰高信噪比分别为约1∶3和1∶10,按1.4.4节供试品取样量和稀释倍数计算,本方法的检出限和定量限分别为2.5 mg/kg和6.25 mg/kg。

#### 2.2.4 回收率

本方法通过向空白样品中添加低、中、高3个水平的特征肽标准溶液,每个水平进行6次平行试验,计算方法回收率。结果见表2,回收率为95.5%~101.9%,各水平平行测定测定结果的相对标准偏差(RSD)为1.1%~3.2%,完全能够满足实际样品的检测需求。

**表 2:** 3个水平下的加标回收率及RSD(*n*=6)

Added/(mg/kg)	Found/(mg/kg)	Recovery/%	RSD/%
40	38.21	95.5	3.2
80	78.55	98.2	1.1
120	122.29	101.9	2.5

#### 2.2.5 精密度

取1.4.3节10 ng/mL的特征肽对照品溶液,重复进样6次,计算定量离子对所对应峰面积的RSD,结果为1.2%,显示仪器精密度良好。

#### 2.2.6 稳定性

取1.4.4节的供试品溶液,置于8 ℃,分别于0、2、4、6、8、16及24 h进样测定,计算定量离子对所对应峰面积的RSD,结果为2.6%,说明供试品溶液中特征肽在8 ℃下24 h内稳定。

#### 2.2.7 重复性

精密称取SV1蛇毒样品6份,每份20 mg,按1.4.4节方法,制备供试品溶液,同上进样分析,结果6份样品的平均含量为102.51 mg/kg, RSD为3.3%,显示本方法重复性良好。

### 2.3 实际样品测定

采用本方法对收集到的6批蛇毒样品进行测定,结果显示,SV1~SV3样品均呈现与对照特征肽EAYNGLPAK色谱保留时间一致的色谱峰,确认其来源于矛头蝮蛇,采用外标法定量,结果显示其特征肽含量范围为102.51~105.40 mg/kg。SV4~SV6样品在对照特征肽色谱保留时间处均未提取到色谱峰,显示未检测到该特征肽,因此为非矛头蝮蛇来源蛇毒。

## 3 结论

本研究针对矛头蝮蛇蛇毒,采用普适性强的酶解条件,对纯化的目标蛋白质进行酶解,获得的目标蛋白特征肽靶向性更强,且避免了其他蛋白质的干扰,数据分析简单且准确性高。为保证方法的实用性和可推广性,本研究进一步针对所选特征肽优化了样品前处理方法、色谱和质谱条件,建立了基于特征肽的超高效液相色谱-串联质谱检测矛头蝮蛇蛇毒种属来源及类凝血酶含量的方法。本方法为追溯蛇毒的物种来源提供了一种快速、稳定、灵敏、特异的检测技术,也可应用于矛头蝮蛇蛇毒、矛头蛇毒血凝酶及其制剂中类凝血酶的含量检测,对于优选蛇毒原料、优化生产工艺、提高产品安全性具有重要意义。
